# From machine learning to multimodal models: The AI revolution in enzyme engineering

**DOI:** 10.1016/j.bidere.2025.100044

**Published:** 2025-08-29

**Authors:** Ziyan Shi, Shuping Xu, Sihan Xue, Kaiming Chen, Yifan Lu, Feiyue Wang, Siyu Long, Yannan Tian, Peng Zhang, Jianing Wang, Yanhui Gu, Junsheng Zhou, Hao Zhou, Shuaiqi Meng, Haiyang Cui

**Affiliations:** a State Key Laboratory of Microbial Technology, College of Life Sciences, Nanjing Normal University, Nanjing, 210097, China; b Ministry of Education Key Laboratory of NSLSCS, Nanjing Normal University, Nanjing, 210097, China; c School of Computer and Electronic Information/School of Artificial Intelligence, Nanjing Normal University, Nanjing, 210097, China; d School of Chemistry and Materials Science, Nanjing Normal University, Nanjing, 210097, China; e College of Marine Science and Engineering, Nanjing Normal University, Nanjing, 210097, China; f Institute for AI Industry Research, Tsinghua University, Beijing, 100084, China; g Department of Systems Biology, School of Life Sciences, Southern University of Science and Technology, No. 1088 Xueyuan Avenue, Shenzhen, 518055, China; h Institute for Biological Electron Microscopy, Southern University of Science and Technology, No. 1088 Xueyuan Avenue, Shenzhen, 518055, Guangdong, China; i State Key Laboratory of Microbial Technology, Shandong University, No. 72 Binhai Road, Qingdao, Shandong, 266237, China

**Keywords:** Machine learning, Enzyme engineering, Enzyme function, Artificial intelligence, Protein language model

## Abstract

Protein engineering is a powerful tool for applications spanning synthetic biology, biocatalysis, and drug discovery. Recent advances in artificial intelligence (AI), from conventional machine learning (ML) algorithms to large-scale pre-trained protein models, have greatly accelerated enzyme engineering field entering a data-driven era. This review provides a guidance map of current enzyme engineering tasks and builds an integrative perspective on AI methods, model types, landmark tasks, and data resources. We begin by delineating the core modeling tasks in enzyme engineering, which include encompassing function annotation, structural modeling, and property prediction and by reviewing recent advances alongside dominant algorithmic frameworks. Next, we outlined the evolution of AI into enzyme engineering, tracing its progression through four stages: classical machine learning approaches, deep neural networks, protein language models (pLMs), and emerging multimodal architectures. Finally, we highlight four trends that are redefining the landscape of AI-driven enzyme design: (i) the replacement of handcrafted features with unified, token-level embeddings; (ii) a shift from single-modal models toward multimodal, multitask systems; (iii) the emergence of intelligent agents capable of reasoning; and (iv) a movement beyond static structure prediction toward dynamic simulation of enzyme function. Together, these developments are paving the way for intelligent, generalizable, and mechanistically interpretable AI platforms poised to synthetic biology.

## 1 Introduction

### 1.1 The central role of enzymes

Enzymes are an important class of biomolecules with catalytic functions, playing indispensable roles in sustaining life [1]. They are essential not only in biological systems but also in a wide array of industries applications [2]. Enzymes are predominantly proteins produced by living cells, with a small fraction being catalytic RNAs. Enzyme often exhibit high substrate specificity and remarkable catalytic efficiency, making them indispensable in various biological and industrial processes. Due to their structural diversity and functional versatility, enzymes hold substantial application value in areas such as synthetic biology, metabolic engineering, and therapeutic development.

In enzymatic reactions, substrates bind to enzymes to form enzyme-substrate complexes, resulting in stabilized interactions and reduced molecular degrees of freedom. The stabilization of the transition-state complex and the associated entropy reduction lower the activation energy, thereby significantly enhancing the reaction rate. A prevailing model for substrate recognition is the induced fit theory, which posits that substrate binding induces conformational changes in the enzyme. This structural rearrangement allows the enzyme to reshape its active site to accommodate the ligand more precisely, facilitating the formation of the enzyme-substrate complex and promoting efficient catalysis.

Catalytic activity is largely determined by specific amino acid residues located at the enzyme's active center, which play a pivotal role in the chemical transformation of substrates into products. The precise regulation of enzyme activity is essential for cellular survival, efficiency, and adaptability. By modulating when, where, and how enzymes function, cells can respond effectively to internal and external cues, maintain homeostasis, and dynamically adapt to environmental fluctuations.

### 1.2 What is enzyme engineering?

Enzyme engineering refers to the process of tailoring enzyme sequences and structures to meet specific functional or physicochemical requirements, with the ultimate goal of generating enzymes optimized for industrial, biomedical, or biotransformation applications. This field primarily involves three complementary strategies: 1.) Directed evolution mimics the process of natural selection by introducing random mutagenesis, such as single-point [3] or saturation mutagenesis [4], followed by high-throughput screening [5] to identify variants with improved performance. 2.) Rational design and semi-rational design utilizes structural and sequence information from known enzymes to guide targeted modifications at specific positions, offering a more informed and efficient approach to enhancing enzyme function. 3.) *De novo* design aims to create entirely new enzyme sequences without relying on any natural enzyme templates, guided by desired properties using molecular modeling, molecular dynamics simulations, or end-to-end deep learning (DL) frameworks. All three strategies focus on the enzyme as the central object of engineering, ultimately aiming to obtain sequences that encode enzymes with the desired functionality. However, when confronting these persistent technical bottlenecks, which include the random mutagenesis of experimental screening, the empirical dependency of rational design, and the complexity of industrial adaptation, traditional enzyme engineering approaches face fundamental challenges. The rapid advancement of artificial intelligence (AI), particularly the deep application of machine learning, deep learning, and generative models in life sciences, presents breakthrough opportunities to address these core issues.

### 1.3 AI for enzyme engineering

Deep learning models can be trained on vast bioinformatics datasets to learn the relationships between protein sequence, structure, and function, thereby providing accurate and efficient guidance for protein design. AI for Enzyme are downstream applications of AI for Protein. Structure prediction has been widely used to support enzyme function characterization by enabling the identification of active sites, substrate-binding pockets, and conformational dynamics related to catalysis.

Traditional model-driven rational design relies on pre-established theoretical models and known rules of enzyme structure-function relationships as its foundation, guiding enzyme modification and design through logical deduction and theoretical computation. The core breakthrough of AI-driven enzyme engineering lies in establishing an integrated *Mining-Design-Validation* triad, which is fundamentally data-driven as it identifies patterns within existing data to predict properties of previously unseen yet analogous inputs. To serve specific biocatalytic tasks (*e.g*., thermostability optimization), researchers typically construct computational models. These models formalize mathematical relationships and algorithmic architectures to generate computational mapping functions, whose essential mission is to abstract the complexity of biological systems into computable quantitative relationships. This enables the prediction or generation of target properties from input data. Specifically, a model establishes a mapping from input variables X (*e.g*., enzyme sequence and structure) to output variables Y (*e.g*., catalytic activity, stability, or selectivity). By learning intrinsic biological mechanisms from training data, models ultimately facilitate the rational design of tailored biocatalytic solutions [6].

AI first aids enzyme mining by identifying potential enzyme sequences with desired features from vast pools of uncharacterized sequences. Leveraging this mined sequence data, machine learning further assists enzyme design by generating novel, previously unobserved enzyme variants with high promise. Distinct from the iterative selection of existing variants in directed evolution, machine learning or deep learning-based design can generate novel and promising variants based on patterns discerned from collected data [7]. The integration of AI technologies, particularly the convergence of deep learning and knowledge graphs, is reconstructing the foundational logic of enzyme engineering.

### 1.4 Scope of the current review

This review aims to provide a systematic and up-to-date summary of AI applications in enzyme engineering, with a particular focus on function-oriented modeling tasks.

Rather than offering an exhaustive survey, we emphasize emerging methodological paradigms and their alignment with practical challenges in enzyme engineering. The review is intended for researchers across computational and biological domains, from those new to AI tools and those aiming to apply AI to enzyme-related problems.

## 2 Functional evaluation and metrics

The criteria for determining an ideal enzyme typically center on improvement in catalytic performance. These include increasing product yield [8], reducing by-product formation [9], or expanding substrate scope from natural to non-natural compounds [10].

When catalytic performance is already satisfactory, the focus may shift to enhancing the enzyme's robustness/stability, which include thermal stability, pH tolerance and reaction selectivity, to guarantee consistent output under industrial or physiological conditions. For example, researchers may improve antibody affinity or stability, boost the fluorescence intensity of a panel of fluorescent proteins, or raise the DNA-cleavage activity of nucleases [6]. Ultimately, the goal is to deliver enzymes whose functional and physicochemical profiles satisfy the stringent demands of downstream applications in synthetic biology, metabolic engineering and biopharmaceutical development.

Determining whether an engineered enzyme meets desired functional and physicochemical specifications requires a combination of quantitative metrics and methodological frameworks. While experimental validation remains the gold standard for evaluating catalytic performance and stability, its scalability is increasingly challenged by the vastness of enzyme sequence space and the expanding scope of mutagenesis efforts [11,12]. Hence, computational modeling has emerged as a powerful and cost-effective alternative, offering rapid and robust insights that accelerate the protein engineering process.

Recent advances in AI-driven experimental workflows have enabled high-throughput measurement of enzymatic activity, product specificity, and biophysical robustness. Techniques such as automated directed evolution, microfluidic screening, and fluorescence-based activity assays, which augmented by machine learning algorithms for candidate prioritization, have significantly improved the throughput and efficiency of wet-lab validation.

An enzyme commission (EC) number is a numerical classification system for enzymes based on the chemical reactions they catalyse. The system was developed by the International Union of Biochemistry and Molecular Biology (IUBMB) to provide a systematic and universally recognized way of identifying and categorizing enzymes. The EC number is a four-part identifier separated by periods (*e.g*., 1.1.1.1), where each digit represents a specific level of enzyme classification like shown in Table .1. Computational methods have become a core tool for enzyme functional evaluation. Their powerful predictive capabilities cover key aspects ranging from EC number assignment, active site localization, to kinetic parameters (such as *K*_*M*_, *k*_*cat*_, and *k*_*cat*_/*K*_*M*_) [2,13,14]. In short, these approaches allow fine-grained functional annotation of enzyme variants even in the absence of experimental labels.

**Table 1:** IUBMB enzyme classes, their general reaction patterns, and typical subclasses.

Enzyme class	General reaction	Characteristics and representative subclasses
**EC 1**		
Oxidoreductases	A_red_ + B_ox_ ⇌ A_ox_ + B_red_	Catalyse oxidation–reduction (electron-transfer) reactions. Examples: oxidases, dehydrogenases, mono-/dioxygenases, peroxidases, *etc.*
**EC 2**		
Transferases	A−B + C → A + B−C	Transfer a functional group from one substrate to another. Examples: methyl-, amino-, acyl- and glycosyl-transferases; kinases (phosphotransferases); sulfur-transferases, *etc.*
**EC 3**		
Hydrolases	A−B + H_2_O → A−H + B−OH	Hydrolytically cleave bonds in the substrate. Examples: proteases, esterases, lipases, glycosidases, nucleases, phosphatases, amidases, peptidases, *etc.*
**EC 4**		
Lyases	A−B ⇌ A + B	Break bonds by mechanisms other than hydrolysis or oxidation, often forming a new double bond; the reverse reaction is an addition. Examples: decarboxylases, deaminases, aldolases, hydratases, synthases, carbon–carbon lyases, *etc.*
**EC 5**		
Isomerases	A−B−C ⇌ A−C−B	Catalyse intramolecular rearrangements (isomerisations). Examples: racemases, epimerases, cis–trans isomerases, mutases, tautomerases, *etc.*
**EC 6**		
Ligases	A + B + ATP → A−B + ADP + P_i_	Join two molecules with concomitant nucleotide triphosphate hydrolysis. Examples: synthetases, carboxylases, peptide ligases, DNA/RNA ligases, *etc.*

In addition to functional annotations, computational tools have shown great success in predicting a range of key enzyme properties, including catalytic activity, thermal and pH stability, selectivity and binding affinity toward substrates or inhibitors. These properties can be inferred from sequence, structure, or a combination of both, using pretrained protein language models, three-dimensional structure encoders, or hybrid models incorporating molecular dynamics simulations.

Taken together, these computational approaches offer a comprehensive evaluation pipeline that complements and accelerates experimental efforts, enabling efficient prioritization of enzyme variants with improved function, stability, and applicability in synthetic biology, metabolic engineering, and biocatalysis.

## 3 From individual-enzyme function modeling to enzymatic pathway design

### 3.1 Single-enzyme-centered modeling

Although enzyme engineering focuses on the design and optimization of individual enzymes, enzymes themselves do not function in isolation. Their catalytic behavior is profoundly influenced by a variety of contextual factors, including the chemical environment, substrate/product identity, cofactor availability, reaction intermediates, protein complex formation, and even the surrounding metabolic network. The modeling centered on the enzyme's environment must consider key variables including pH, temperature, and solvent composition, all of which can impact enzyme activity and stability. Resources like UniKP not only provide condition-specific kinetic parameters(*e.g*., *k*_*cat*_ values under varied pH and temperature), but have also been applied in practical enzyme function optimization tasks. For instance, UniKP was used to mine novel tyrosine ammonia lyase (TAL) enzymes with higher catalytic activity, and to guide directed evolution of RgTAL variants. This resulted in the discovery of TAL variants with up to 3.5-fold improved catalytic efficiency (*k*_*cat*_/*K*_*M*_), demonstrating its utility in real-world enzyme engineering efforts [15].

When focusing on the substrate, modeling tasks extend from enzyme sequence design and function prediction to small molecule generation. Relevant downstream tasks include reaction equation completion, substrate similarity search, and the design of covalent or non-covalent inhibitors targeting specific substrates.

Product-focused modeling involves predicting primary products, minimizing by-product formation, and evaluating enzyme tolerance to product feedback inhibition [16], highlight the integration of structural biology, molecular dynamics, and biochemical validation.

When modeling focuses on cofactors, such as NADP(H), FAD, or metal ions, the objective expands beyond their mere identification [[17], [18], [19]]. Tasks include predicting cofactor binding specificity, optimizing intracellular cofactor regeneration and recycling strategies, and maintaining cofactor availability through dynamic concentration balancing across enzymatic steps.

Inverse folding, which refers to identifying sequences that fold uniquely into a target structure, represents a key modeling task when structural specificity is essential. Unlike conventional structure prediction, which maps sequences to structures, inverse folding operates in the reverse direction, where compatible amino acid sequences are generated for a given structural scaffold. This paradigm is particularly critical in enzyme engineering, since the objective is often to preserve catalytic motifs while at the same time optimizing overall stability and activity. Recent advances have further extended inverse folding for functional prediction, as exemplified by the Boltzmann Alignment technique, which adapts pre-trained inverse folding models in order to estimate binding free-energy changes (ΔΔ G). By incorporating thermodynamic principles, including the Boltzmann distribution and Bayes’ theorem, this approach achieves both supervised and unsupervised state-of-the-art performance in predicting mutational effects on protein–protein interactions [20].

Enzyme complexes introduce tasks such as protein–ligand, protein–protein, or protein–nucleic acid structure prediction. This aligns with advances in AI-driven complex modeling (*e.g*., AlphaFold-Multimer) for multi-subunit or transient interactions.

### 3.2 From enzymes to pathways

Enzymes often function in concert, particularly in metabolic pathways or synthetic cascades. In this context, modeling shifts from optimizing single-enzyme properties to balancing multi-enzyme coordination, involving expression levels, catalytic efficiency, and metabolite flux.

Tasks such as pathway optimization, cell factory design, and virtual cell modeling emerge at this field. For instance, human gut *Bacteroides* degrade dietary pectic glycans via coordinated multi-enzyme systems [21]. Similarly, strategies like iMECS [22] demonstrate *in vitro* co-expression and cofactor self-cycling in lignin valorization. There is also the design of RBS libraries using deep learning tools to optimize the metabolic pathway for the production of limonene compounds in *E.coli* [23]. These multi-enzyme approaches reflect the convergence of enzyme engineering with synthetic biology, systems biology, and biomanufacturing.

When centered on reaction pathways, modeling includes pathway design, retro-synthesis of enzyme-catalyzed steps, and multi-step reaction optimization. Expending this perspective to the metabolic network level, modeling tasks include pathway analysis, network-based pathway prediction, and evaluation of thermodynamic and kinetic feasibility. Studies [24,25] highlight how enzyme-centric modeling can improve the accuracy of constraint-based metabolic predictions. The Institute of Engineering Technology at the Chinese Academy of Sciences, based on the EcoETM model, treats enzymes as micro-compartments and has established a metabolic network model that incorporates spatial constraints of enzymes. This work significantly improves pathway prediction accuracy and the reliability of engineering targets.

Ultimately, the transition from single-enzyme function modeling to multi-enzyme system design underscores a paradigm shift toward systems-level control, where the emergent behavior of coordinated enzymes must be understood, predicted, and optimized holistically.

## 4 Modeling of enzyme structure, properties, environment and functions

As most enzymes are proteins, AI applications in enzyme engineering naturally build upon advance in AI for protein, with particular emphasis on modeling tasks that directly related to enzymic function. Artificial intelligence plays a central role in predicting enzyme function by learning complex sequence–structure–activity relationships. Function prediction encompasses a broad array of tasks that include identifying substrate specificity [26], analyzing activation mechanisms [27], and modeling catalytic efficiency [28]. These involve detailed protein–ligand interaction modeling, such as annotating binding sites [29], calculating substrate binding affinities using metrics like compound-protein interactions (CPI) scores [30], predicting reaction activation energies [31], and conducting virtual screening of small-molecule agonists and inhibitors [32,33]. Besides modeling single-step catalytic reactions, enzyme functions are also classified and represented by gene ontology (GO), encompassing Biological Process (BP), molecular function, and cellular component (subcellular localization).

For enzyme structure modeling, AI methods have achieved breakthroughs in atomic-level predictions. Models such as AlphaFold2 [34], AlphaFold3 [35], ESM-3 [36], Rosetta [37], and Chai-1 [38] enable the prediction of 3D atomic structures for enzymes and their complexes with substrates.

AI is also extensively applied in predicting key physicochemical properties of enzymes. For example, models like ProtSSN [6] are used to estimate thermostability. Other deep learning models can predict pH stability, catalytic activity, overall stability, selectivity, and binding affinity, assisting in the screening of optimal enzyme variants.

Additionally, several specialized modeling directions are actively being explored. Mechanistic analyses that focusfocusing on substrates or metal ions offer deep insights. For example, in studies on *human carbonic anhydrase II (CA II)*, high-pressure X-ray crystallography was used to resolve the coordination between CO_2_ and metal ions, followed by QM/MM simulations to elucidate the effect of different divalent transition metal ions on enzyme activity [39].

The thermodynamic rationale behind enzymatic catalysis often involves lowering activation energy via substrate binding. Thus, computing enzyme–substrate binding energies provides a thermodynamic basis for catalytic property prediction [40].

Allosteric modulation modeling is also critical. By integrating TR-HT-SAXS techniques, researchers have screened small-molecule allosteric modulators that induce conformational transitions in oxidoreductase AIF. SAXS data were used to construct a multi-dimensional dataset that identified promising modulatory lead compounds [41].

Finally, AI is critical for predicting drug resistance in enzyme variants. A recent model named *Dr. Kinase*, developed by a domestic research team, employs a deep hybrid learning framework to predict four key drug-resistance hotspots in kinases (gatekeeper site, G-loop, *α*C-helix, and A-loop), achieving an AUC up to 0.99 on independent test sets [42]. The aforementioned models and their performance metrics, covering functional prediction, structure modeling, property prediction, and various specialized modeling tasks (such as mechanistic analysis, binding energy calculation, allosteric modulation modeling, and resistance prediction), will be specifically analyzed and compared in Section 7 (Tasks on Enzyme Engineering).

## 5 Datasets

In AI-driven protein and enzyme engineering, structured data resources form the backbone of the field by providing biomolecular descriptors, including sequence information, three-dimensional structures, functional annotations, and biochemical properties. Broadly, these resources can be categorized into two categories.

1. Continuously updated databases such as the PDB and UniProt, which integrate experimentally determined protein structures, sequences, and functional annotations in real time.
2. Task-optimized datasets such as UniRef90 and USPTO-50K, which are curated, standardized, and split specifically for machine-learning applications.

Both types of resources are pivotal for training robust models. The breadth of large-scale bioinformatics data determines how thoroughly a machine-learning model can explore protein sequence space and structure–function relationships. Meanwhile, data accuracy, consistency, and annotation quality directly affect the reliability of predictions. Specialized databases targeting particular biological processes, such as enzyme kinetics, protein–protein interactions, and conformational dynamics, make function-focused design possible.

To ensure that biological data can be effectively translated into computational resources, key quality criteria must be met. This include completeness, logical consistency, timely updates, and controllable bias. Meeting these standards ensures that raw biological information is effectively transformed into computational resources, providing deep-learning frameworks with a knowledge base that is both biologically sound and practically useful for engineering.

### 5.1 Key databases

Current mainstream databases(Table .2 and Table .3), according to their core function, can be divided into four categories: (1) structure databases. This type of database provides atomic-level three-dimensional coordinate information, with typical representatives including PDB and AlphaFold DB, the former providing training benchmarks for structure-prediction models, the latter accelerating the functional exploration of monomeric proteins; (2) sequence and function databases. Among them, UniProt, containing more than 220 million entries, is the foundation for pre-training of protein structure and function models, while BRENDA, by virtue of its manually annotated enzyme-kinetic parameters *K*_*M*_,*k*_*cat*_, *k*_*cat*_/*K*_*M*_, becomes a key reference for enzyme-engineering optimization; (3) interaction databases. This type of database focuses on molecular-interaction mechanisms, PDBbind includes more than 27,000 protein–ligand complexes and, through rigorous quality control, provides a gold standard for binding-affinity prediction, whereas Binding MOAD, containing 5331 high-quality complexes, assists drug design with binding-strength data spanning 13 orders of magnitude; (4) specialized functional databases. For example, ChEMBL drives substrate-specificity design, and the 803 intrinsically disordered protein experimental data in DisProt support research on conformational dynamics. It is worth noting that these databases present certain limitations, such as CATH lagging behind newly added PDB entries, resulting in update latency; KinaseMD lacks clinical-response data, with coverage that is not sufficiently complete, so bias-correction strategies need to be introduced in model construction.

**Table 2:** Overview of representative databases.

Database	Property	Size	Advantages	Disadvantages	Location	Reference
AlphaFill	Ligand structure transplantation	2694 ligands transplanted, covering ∼95 % of ligand occurrences in PDB	Includes all cofactors; Optimized for high-frequency ligands	Potential distortion of interactions; Updates depend on new PDB releases	https://alphafill.eu	[34]
AlphaFold	AI-predicted protein structures	∼214 million structures (2025, covering nearly all catalogued organisms)	Covers virtually all known proteins; Accelerates drug discovery for neglected diseases; Free access	Predictions may lack experimental validation; Limited accuracy for dynamic protein conformations	https://alphafold.ebi.ac.uk	[43]
Azure Cosmos DB	Globally distributed multi-model database	Service-based	Multi-model support; Automated global data distribution	High operational costs; Lacks dedicated tools for biological data	https://azure.microsoft.com/products/cosmos-db	[44]
Binding MOAD	High-quality protein–ligand complexes	5331 complexes; 1780 protein families	Non-redundant dataset; Binding affinity spans 13 orders of magnitude	Only 26 % complexes include binding data; Inconsistent crystallographic resolutions	http://bindingmoad.org	[45]
BRENDA	Enzyme functional data	∼84,000 manually annotated enzymes; ∼160,000 text-mined entries	Comprehensive enzyme coverage (11,000 organisms); Includes mutants and kinetic parameters; Detailed reaction annotations	Inhomogeneous data quality; Requires stricter quality control due to multiple sources	https://www.brenda-enzymes.org	[46]
CATH	Protein domain classification	∼300,000 domain entries (v4.3)	Hierarchical functional annotation; Integrates structural and evolutionary data	Updates lag behind PDB; Limited coverage of non-globular proteins	http://www.cathdb.info	[47]
ChEMBL	Manually curated bioactive molecules	>2.4 M compounds; >1.8 M assays	Integrates chemical/genomic/bioactivity data; Drug-development-oriented design	Non-standardized bioactivity data; Limited natural-product coverage	https://www.ebi.ac.uk/chembl	[48]
DCDB	Drug-combination screening	448,555 combinations; >2887 drugs	Integrates experimental/literature/failed combinations; Provides training sets for predictive models	Limited cell-line sources (human); Incomplete mechanistic annotations	http://dcdb.chemnetbase.com	[49]
DisProt	Intrinsically disordered proteins	803 entries	Experimentally verified regions; CSV/JSON download options	Small dataset size; Functional annotations depend on literature	https://disprot.org	[36]
GDB	Enumeration of small organic molecules	166.4 billion molecules (up to 17 atoms)	Covers all possible stable organic molecules ≤17 atoms; Enables virtual screening for novel drug scaffolds; Free access	No experimental data (computed structures only); Excludes radicals/ions; Limited to small molecular weight range	https://gdb.unibe.ch	[50]
GEO	Public gene-expression repository	>150 k studies (2025)	Four-level ID system; Enables differential expression/pathway analysis	Inconsistent raw-data formats; Partial metadata missing	https://www.ncbi.nlm.nih.gov/geo	[51]
JGI-IMG	Microbial genome annotation	>80,000 bacterial genomes	Integrates CRISPR/ncRNA annotations; Uses COG/KEGG functional libraries	Sparse eukaryotic coverage; Delayed annotation updates	https://img.jgi.doe.gov	[36]
KEGG	Metabolic pathways and networks	∼90 reference metabolic pathways; 83 ortholog groups	Graphical pathway visualization; Links genomes to cellular functions; Supports *in silico* metabolic analysis	Pathway curation may contain biases; Limited mechanistic-validation data	https://www.kegg.jp	[52]
KinaseMD	Kinase mutations & drug responses	679,374 somatic mutations; 251,522 rewiring events; 390,460 drug-response records for 547 kinases	Integrates somatic-mutation and drug-response data	Incomplete kinase-type coverage; Limited clinical response data	http://kinasemd.org	[53]

**Table 3:** Overview of representative databases.

Database	Property	Size	Advantages	Disadvantages	Location	Ref
MetaCyc	Experimentally elucidated metabolic pathways	3006 pathways; 18,124 metabolites	Supports metabolic-pathway prediction; Covers primary and secondary metabolism	Annotations lag literature updates; Missing pathways for some microbes	https://metacyc.org	[25]
MGnify	Metagenomic protein analysis	>1 B sequences; 437 k analyses	Provides HMMER homology search; Non-redundant protein set	Missing functional annotations; Variable assembly quality	https://www.ebi.ac.uk/metagenomics	[54]
OAS	Antibody-sequence repository	∼1.5 B sequences (80+ studies)	Enables AI-driven antibody humanization; Includes immune-state annotations	Sequence errors require correction; Limited species coverage	http://opig.stats.ox.ac.uk/webapps/oas	[36]
PDB	Experimentally determined macromolecular structures	>200,000 structures (2025)	Largest repository of 3-D biological structures; Rich metadata and validation reports	Incomplete membrane-protein coverage; Format-standardization issues; Missing dynamic-transition data	https://www.rcsb.org	[12]
PDBbind	Protein–ligand binding affinity	>27,000 complexes (2024)	Strict quality control in refined set; High-reliability data for molecular docking	Potential omission of abnormal complexes; Limited membrane-protein coverage	http://www.pdbbind.org.cn	[55]
Pfam	Protein-family classification	19,632 families (v35.0)	Accurate HMM-based alignment; Compatible with hmmsearch	Simplified functional annotations; Occasional domain-boundary inaccuracies	http://pfam.xfam.org	[56]
SABIO-RK	Biochemical reaction kinetics	>700,000 kinetic parameters	Provides kinetic equations and experimental conditions; Supports biochemical-network modeling	Heterogeneous data sources; Manual validation needed for experimental consistency	https://sabio.h-its.org	[46]
SCOP	Protein-structure classification	>5000 structural families	Hierarchical classification; Systematic functional annotation	Updates lag behind PDB; Imperfect membrane-protein classification	http://scop.mrc-lmb.cam.ac.uk	[57]
Single Cell Portal	Single-cell research-project database	4,152,655 single cells from 169 studies	Dual search modes (Study/Gene); Interactive visualizations; Direct linkage to GEO; Partial raw-data download	Partial datasets restrict download; Visualization relies on predefined workflows	https://singlecell.broadinstitute.org	[58]
T3DB	Toxins and toxic compounds	∼3900 toxins with chemical/biological data	Detailed toxicological profiles (mechanisms, targets); Cross-references to pharmacological databases	Sparse data for rare toxins; Limited experimental kinetic parameters	http://www.t3db.ca	[59]
UniProt	Protein sequences and functional annotations	>220 M entries (Swiss-Prot reviewed; TrEMBL unreviewed)	High-quality manual curation (Swiss-Prot); Non-redundant clustering (UniRef); Integrated analysis tools	Redundant annotations in TrEMBL; Limited reliability for unreviewed entries	https://www.uniprot.org	[38]
UniRef	Non-redundant protein sequences	Clustered from UniProtKB (100 %/90 %/50 % identity)	Effectively reduces redundancy; Optimized clustering algorithms	Low-similarity sequences may be excluded; Ambiguous cluster boundaries	https://www.uniprot.org/uniref	[38]
ZINC	Commercially available compounds	Tens of billions of molecules	Free access; Pre-computed properties (conformations, cLogP); Scalable similarity search via CartBlanche	Molecular diversity may plateau at ultra-large scales; Computational cost for docking increases	https://cartblanche22.docking.org	[60]

### 5.2 Key datasets

Datasets oriented toward machine learning(Table .4 and Table .5), through meticulous processing, turn raw databases into computer-useable input data, and datasets can be divided into three categories: (1) sequence-clustering datasets. Non-redundancy is achieved by identity-similarity thresholds, for example UniRef100 [61], UniRef90 [62], and UniRef50 [62], which respectively merge completely identical sequences, sequences with similarity over 90 %, and sequences with similarity over 50 %, forming a progressively compressed database structure and, through redundancy reduction, improving the efficiency of remote-homology detection; (2) functional-prediction datasets. This kind of dataset focuses on quantifying specific biological properties, VenusMutHub [63] integrates multidimensional scores of stability and adaptability to support mutation-effect modeling, and the SABIO-RK [46] dataset, which registers more than 700,000 kinetic parameters, empowers optimization of enzyme catalytic efficiency through annotation of reaction equations and experimental conditions. The Deep Mutational Scanning (DMS) [64] dataset collection provides 12 distinct protein activity landscapes spanning Viral spiking proteins, RNA-directed nucleases, DNA-binding proteins, RNA-binding proteins and kinases, enhancing the performance of the EVOLVEpro model for predicting the directed evolution of proteins. The GB1 [65] dataset specifically quantifies IgG-binding affinity for over 500,000 double-mutant variants of protein GB1; (3) task-benchmark datasets. This kind of dataset provides a unified yardstick for algorithm evaluation, the TS500 [66] dataset contains 500 pairs of protein structures and becomes a testing benchmark for fold-recognition models, USPTO-50K [67] collects about 50,000 chemical reactions and standardizes the evaluation workflow of retrosynthetic-route prediction. In addition, there are experimentally validated datasets, such as MISATO [68], which integrates 19,443 quantum-mechanically optimized ligand conformations to give empirical support to AI-design results.Notably, TrpB [69] contains 160,000 sequences of mutations at four sites of the active site of the tryptophan synthase (TrpB) *β* subunit, capturing significant non-additive effects of substitutions on catalytic function. This kind of data, by comparing computational predictions with actual experimental results, helps narrow the gap between the two and is a key bridge that propels protein design from computer simulation toward practical application.

**Table 4:** Overview of representative datasets.

Dataset	Property	Size	Advantages	Disadvantages	Location	Reference
BaseData	Genomic & protein sequences	>9.2T genomic tokens; 9.8B protein sequences	Rigorously screened new species sequences; Optimized for large-scale AI training	Non-public database; Restricted access permissions	Undisclosed	[70]
BFD	Large-scale protein sequence database	2.2B sequences; 1.8 T data	Massive training dataset; Ideal for protein structure prediction	High data redundancy; Maintenance depends on third parties	http://bfd.mmseqs.com	[47]
DrugBank	Bioactive molecules & drug targets	13,791 drug entries; 5236 non-redundant proteins	Integrates drug structures/targets/pathways; Advanced search and visualization tools	Delayed updates for small-molecule drugs; Incomplete coverage of biotech drugs	https://go.drugbank.com	[71]
Uniclust30	Protein sequence clustering	Clustered at 30 % identity	Better annotation consistency than UniRef; MMseqs2-accelerated clustering	May exclude low-similarity sequences; Fixed clustering threshold	https://uniclust.mmseqs.com	[72]
TS50	Protein structure alignment benchmark	50 high-quality protein pairs	Standardized test set; Focused evaluation of alignment tools	Small sample size; Limited structural diversity	http://zhanggroup.org/TM-align	[66]
TS500	Protein structure alignment benchmark	50 high-quality protein pairs	Standardized test set; Focused evaluation of alignment tools	Small sample size; Limited structural diversity	http://zhanggroup.org/TM-align	[66]
DIP	Protein-protein interactions	80K interactions	High-quality manual curation; Integrates multiple experimental methods; Cross-references to other databases	Limited coverage of non-model organisms; Updates slower than automated resources	https://dip.doe-mbi.ucla.edu	[73]
HPRD	Human protein annotations	30K proteins (discontinued)	Comprehensive PTM annotations; Detailed disease associations; Expert-curated entries	No longer maintained (discontinued 2013); Limited to human proteins	http://hprd.org	[74]
MISATO	ML dataset for protein–ligand complexes	QM: 19,443 ligands; MD: 16,972 structures	Integrates QM/MD simulation data; Includes PyTorch benchmark models	Limited simulation duration (10 ns); Partial complexes lack validation	https://misato-project.org	[68]
STRING	Protein-protein interaction networks	67.6M proteins; 2031 organisms (v12.0)	Integrates genomic context and text mining; Confidence scoring system; Interactive network visualization	Indirect associations may introduce noise; Computational predictions require experimental validation	https://string-db.org	[75]
USPTO-50K	USPTO-50K	50K reactions	Standard benchmark for reaction prediction; Well-balanced class distribution; Preprocessed for ML pipelines	Limited to single-product reactions; Contains patent-specific biases	https://github.com/pandegroup/reaction-prediction	[67]
VenusMutHub	Protein mutation effects	>500K mutations	Integrates stability/fitness/function scores; Machine-learning ready format; Unified annotation system	Limited to engineered mutations; Experimental validation coverage varies	http://venusmut.bb.iastate.edu	[63]

**Table 5:** Overview of representative datasets (continued from Table 4).

Dataset	Property	Size	Advantages	Disadvantages	Location	Reference
CATH 4.2	Protein structure classification	235K domains	Hierarchical domain annotation; Stable version for reproducibility	Outdated structural coverage; Missing recent PDB entries	http://www.cathdb.info/version/v4_2_0	[76]
CATH 4.3	Protein structure classification	300K domains	Updated structural coverage; Improved non-globular protein annotation	Membrane protein annotation still limited; Occasional misclassifications	http://www.cathdb.info	[76]
UniRef50	Non-redundant protein sequences	50 M clusters	Maximizes sequence diversity; Ideal for remote homology detection; Reduces search space by 70	Low annotation specificity; Potential inclusion of non-homologous sequences; Fixed 50	https://www.uniprot.org/uniref	[62]
UniRef90	Non-redundant protein sequences	100 M clusters	Balanced diversity/specificity; Optimized for functional annotation transfer; 80	Excludes divergent orthologs; Limited for structural prediction; Cluster boundaries may blur functional domains	https://www.uniprot.org/uniref	[62]
UniRef100	Non-redundant protein sequences	220 M clusters	Full-resolution clusters; Preserves isoform variants; Best for precise sequence identity matching	Highest redundancy; Large computational overhead; Contains fragments and synthetic sequences	https://www.uniprot.org/uniref	[61]
DMS	Protein sequences with deep mutational scanning	12 deep mutation scanning datasets covering various proteins	Maximizes model generalizability; Covers diverse protein functions (viral spikes, nucleases, DNA/RNA-binding, kinases)	Heterogeneous data formats; Requires cross-dataset normalization	Undisclosed	[64]
GB1	Protein-protein interaction (binding to IgG)	>500K variants (2-point mutations)	Comprehensive coverage of all 2-point mutations in 55-residue sequence; High-throughput RNA display selection	Excludes 1-point mutation data; Limited to IgG binding context	https://mavenn.readthedocs.io	[65]
TrpB	Enzyme activity measurements of combinatorial mutations in tryptophan synthase	160K sequences (4-site mutations)	Combinatorially complete epistasis mapping; High-throughput activity profiling; Engineered parent variant (Tm9D8∗) for non-natural context	Limited to four mutation sites; Background restricted to specific variant	https://doi.org/10.1101/2024.06.23.600144	[69]

## 6 AI modeling paradigms

The technologies of AI in enzyme engineering have evolved significantly, progressing from early machine learning method based on statistical analysis to modern deep learning architectures. More recently, the field has advanced towards pre-trained models, large-scale multimodal models, and agent workflows that incorporate general large language models (LLMs) from the NLP domain.

**Fig. 1:**
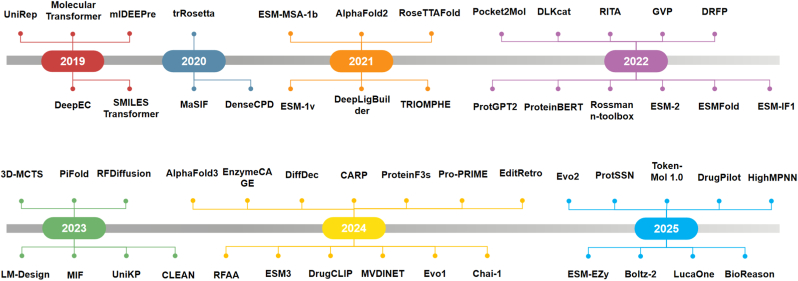
**Representative models in the AI for Enzyme**: A chronological overview of representative models developed for AI-driven enzyme research from 2019 to 2025. Each year is represented by a color-coded node along the timeline, with models plotted according to their initial publication or release date. The timeline captures the evolution from early sequence-based models (e.g., DeepEC, UniRep) and structural predictors (e.g., trRosetta, AlphaFold2), to increasingly sophisticated architectures such as diffusion models (e.g., RFDiffusion), language model variants (e.g., ESM series), and integrated design pipelines (e.g., LM-Design, DrugPilot, TokenMol 1.0). The figure illustrates the rapid growth of this field, highlighting trends toward pre-trained protein models, structure-based generation, and multi-modal enzyme design.

Topics such as protein substitution effect [63], machine learning applications in enzyme engineering [7], AI-driven *De novo* enzyme design [2], and deep learning-guided dynamic protein design [77] have been reviewed. However, in recent years (2019–2025), the rapid development of LLMs in AI has further advances in protein language models and large multimodal, multitask foundation models within the AI for Protein field, even giving rise to efforts such as BioReason that combine biological foundation models with general LLMs. Representative models in the AI for Enzyme domain and their publication years are shown in Fig. 1.

### 6.1 Traditional machine-learning and deep-learning models

**Fig. 2:**
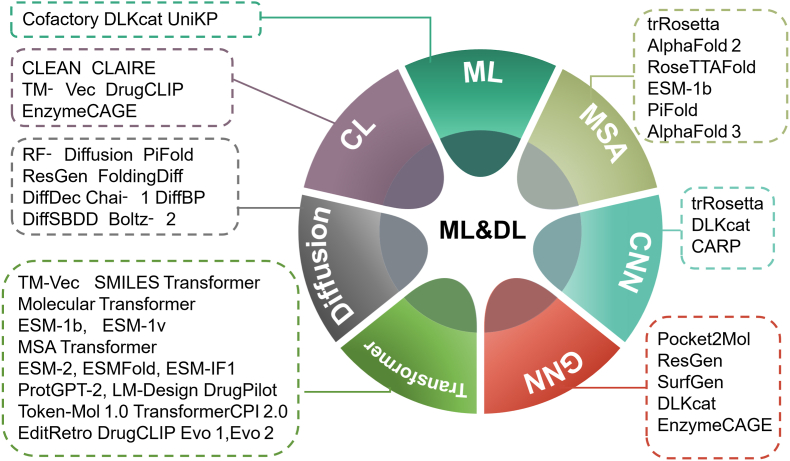
Representative models based on machine learning(ML) and deep learning(DL) architectures. Models are categorized according to their core algorithmic paradigms and learning approaches. ML: Machine Learning; CL: Contrastive Learning; Diffusion: Diffusion Models; Transformer: Transformer Architectures; GNN: Graph Neural Networks; CNN: Convolutional Neural Network; MSA: multiple sequence alignment.

Before the advent of large-scale pretrained models, numerous machine learning and deep learning models had been applied in the field of enzyme engineering. Based on their technical frameworks, these models can be categorized into machine learning-based models, MSA-based methods, CNN-based models, graph neural network (GNN)-based models, Transformer-based models, diffusion-based models, and contrastive learning-based models. Representative works for each category are illustrated in Fig. 2.

#### 6.1.1 Machine-learning-based models

**Fig. 3:**
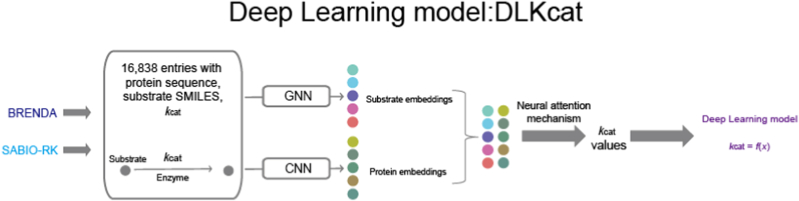
**DLKcat framework**: Model training data is sourced from curated kinetic databases: BRENDA and SABIO-RK. 16,838 entries containing protein sequences, substrate SMILES(Simplified Molecular Input Line Entry System) and experimentally measured Kcat. The model takes two primary inputs: Enzyme and Substrate. In model architecture and data processing, a dual graph neural network (GNN) encoder is utilized. Substrate Embeddings: A GNN processes the substrate's molecular graph (derived from SMILES) to generate a numerical embedding vector capturing its chemical features. Protein Embeddings: A separate GNN processes the enzyme's sequence/structure implied to generate an embedding representing its functional and structural features. The combined enzyme and substrate embeddings are fed into thedeep learning model core to predict the *k*_*cat*_ values.

**Fig. 4:**
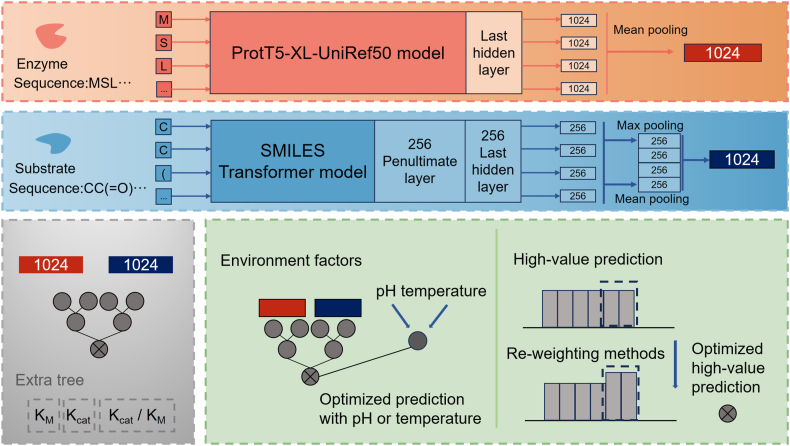
**Overview of UniKP** architecture. Enzyme sequences encoded into 1024-dimensional embeddings via the ProtT5-XL-UniRef50 protein language model; Substrate sequence transformed into 1024-D feature vectors using a SMILES Transformer; UniKP adopts an Extra Trees regression framework to predict *K*_*M*_,*k*_*cat*_, *k*_*cat*_/*K*_*M*_; Environmental factors (pH/temperature) converted into 1024-D representations. All pathways apply mean pooling to consolidate features while preserving dimensionality. The fused features drive two innovations: Condition-specific optimization and High-value prediction enhancement.

In the field of protein design, machine learning (ML) plays a vital role in uncovering the complex relationships among sequence, structure, and function [78]. Instead of focusing on individual models, ML applications in this domain are typically categorized by task types such as classification, regression, clustering, and dimensionality reduction [79]. Common models like Support Vector Machines (SVM) [80], Random Forests (RF) [81], Decision Trees (DT) [82], K-means [83], and neural networks [79] are employed depending on the specific task. For example, classification models are used to assign protein sequences to functional categories or structural classes [84]; regression models predict continuous values such as stability scores, enzyme activity, or binding affinity; clustering methods help identify patterns within protein families or unexplored sequence spaces; and dimensionality reduction techniques like PCA [85] or t-SNE [86] assist in visualizing high-dimensional representations and simplifying features. As protein datasets grow in size and complexity, traditional ML models continue to be valuable, especially in data-scarce or well-defined scenarios, where they often serve as lightweight alternatives or pre-analysis tools complementing deep learning approaches. Machine learning models have been widely used in protein engineering research. The methodological evolution can be best understood by examining representative approaches. At the most basic level, early hybrid modeling techniques such as Cofactory [87] combines a hidden Markov model (HMM) with an artificial neural network (ANN) to predict an enzyme's cofactor preference. DLKcat [88] employs a Bayesian deep-learning framework to predict the catalytic turnover number (*k*_*cat*_) of enzymatic reactions(Fig. 3). UniKP [15] adopts an Extra-Trees regression framework that jointly models protein and substrate representations, extracted respectively from ProtT5 and the SMILES Transformer,to predict key kinetic parameters, including the Michaelis constant (*K*_*M*_), the turnover number (*k*_*cat*_), and the catalytic efficiency (*k*_*cat*_/*K*_*M*_)(Fig. 4).

The choice between traditional machine learning (ML) models and deep learning (DL) approaches depends on multiple factors, such as dataset scale, data quality, task complexity, and the generality of the modeling goal. Traditional ML models like Support Vector Machines (SVM), Random Forests (RF), and Decision Trees (DT) are generally more suitable for small to medium-sized datasets with clearly defined, human-engineered features. These models are relatively easy to train, require less computational resources, and often provide higher interpretability, which is crucial in applications where transparency and explainability are important. Moreover, traditional ML methods tend to be more robust in scenarios where data is noisy, sparse, or exhibits clear domain-specific patterns that can be captured by hand-crafted features. In contrast, deep learning models such as convolutional or transformer-based neural networks are advantageous when dealing with large-scale, complex datasets. They are capable of automatically learning rich, hierarchical representations from raw inputs such as protein sequences, molecular graphs, or multimodal data, and excel in capturing highly non-linear relationships. DL models are especially powerful in tasks that require generalization across diverse sequence spaces or the integration of heterogeneous data types. However, they typically demand more extensive data, higher computational budgets, and careful regularization to prevent overfitting. While traditional ML models remain strong candidates in data-scarce, well-defined tasks, DL approaches have become increasingly dominant in protein engineering due to their scalability and expressive capacity. As such, these two paradigms should be viewed as complementary, with model selection guided by practical considerations such as available data volume, feature quality, and the complexity of the biological question at hand.

#### 6.1.2 MSA-based methods

In protein engineering, multiple-sequence-alignment (MSA)–driven models explicitly exploit co-evolutionary signals in homologous sequences to turn *sequence covariation* into precise structural or functional knowledge. Early trRosetta used residual CNNs to parse MSAs and fed the predicted distance or orientation constraints into Rosetta for folding [89]. AlphaFold-2 followed with the Evoformer, a recycling attention block that iteratively exchanges information along both the sequence and position axes of an MSA, achieving atomic-level accuracy [90]. Its open-source counterpart RoseTTAFold shuttles information across 1-D MSA, 2-D co-evolution maps and 3-D coordinates via a “tri-track” network [91]. ESM-MSA-1b demonstrated that a large Transformer can infer contact maps directly from MSAs by masked-language pre-training [92]. More recently, PiFold replaced the Evoformer with an SE(3) diffusion process, greatly accelerating MSA-conditioned folding [66], while AlphaFold 3 fused the MSA pathway with small-molecule and nucleic-acid representations to predict protein–ligand complexes [35].

#### 6.1.3 CNN-based models

CNN model have also shown remarkable promise in protein modeling. For example, trRosetta [89] employs residual convolutions to infer pairwise distance maps, markedly enhancing structure prediction. DLKcat [88] encodes enzyme protein sequences with a CNN to obtain protein embeddings, concatenates these with substrate embedding vectors, and finally uses a neural attention mechanism to predict *k*_*cat*_ values (Fig. 3). Futhermore, experimental work on CARP [93] shows that, in pre-training tasks, CNNs are competitive with Transformers, while their resource requirements scale linearly with sequence length. More importantly, CNNs remain competitive, and at times even superior, in downstream evaluations, including structure prediction, zero-shot mutation-effect prediction, and out-of-distribution generalization [93]. These empirical results indicate that a deeper understanding of protein-sequence pre-training will require disentangling the influences of model architecture and pre-training objectives.

#### 6.1.4 Graph neural network (GNN)-based models

GNN-based models offer another powerful paradigm. For instance, Pocket2Mol [94] constructs a 3-D pocket graph whose nodes are coarse-grained pocket-surface points annotated with atom type, solvent-accessible surface area (SASA) and electrostatic features, while the edges encode Euclidean distances. An SE(3)-equivariant message-passing network embeds this pocket graph, and the resulting latent vector conditions an autoregressive decoder that sequentially places ligand atoms in 3-D space.

ResGen [95] likewise adopts an SE(3)-equivariant multi-scale generative model to capture protein–small-molecule interactions. Similarly, SurfGen [96] integrates topological and geometric information: its Geodesic-GNN module learns protein-surface topology, whereas its Geoattn-GNN module learns interactions in molecular space. Besides, DLKcat [88] encodes substrate SMILES strings with a graph neural network (GNN) to obtain substrate graph embeddings, concatenates these with protein sequence embeddings, and ultimately predicts *k*_*cat*_ values via a neural attention mechanism (Fig. 3). Furthermore, EnzymeCAGE [97] combines local pocket-level representations generated by GNNs with global enzyme-level features derived from the ESM-2 protein language model.

#### 6.1.5 Transformer-based models

Transformers have emerged as the workhorse of AI-driven enzyme engineering. For example, TM-Vec [98] employs a dual-tower Transformer encoder to map each of two protein sequences to a fixed-length vector, and uses the cosine distance between the vectors to directly regress their structural-similarity metric, the TM-score.

Transformer architecture first entered chemistry through the SMILES Transformer and the Molecular Transformer, which demonstrated that self-attention can capture reaction syntax directly from string representations of molecules and chemical equations [99,100]. Soon afterwards the protein field embraced the same idea: Meta FAIR's ESM family showed that very large language models trained on raw sequences or multiple-sequence alignments could recover long-range couplings and functional signals. ESM-1b and ESM-1v deliver zero-shot variant-effect prediction, the MSA Transformer encodes co-evolution explicitly along sequence and position axes, ESM-2 scales the masked-language objective to 3–15 B parameters for state-of-the-art single-sequence structure and function inference, ESMFold attaches an SE(3) geometric head to the ESM-2 encoder for end-to-end folding, and ESM-IF1 tackles inverse folding with a GVP-enhanced Transformer [54,92,[101], [102], [103]].

On the generative side, ProtGPT-2 and LM-Design can sample libraries of *natural-like* sequences that are later triaged by structure prediction [104,105]. Token-Mol 1.0 unifies conformation generation, property prediction and pocket-conditioned ligand design through 3-D tokenisation followed by a Transformer backbone [106]. TransformerCPI 2.0 treats a protein sequence as natural language and directly produces a matching SMILES string for the ligand partner [107], while EditRetro applies a Transformer that edits reaction graphs step by step to solve retrosynthesis problems and thus suggests metabolic or synthetic routes relevant to enzyme redesign [108].

A newer wave couples Transformer language models with retrieval, physics-based docking or reinforcement learning modules. DrugCLIP pairs a pocket Vision Transformer with a ligand GNN under a contrastive objective, and systems such as Evo 1, Evo 2 and DrugPilot wrap large protein LLMs with agent-style tool calls to support genome-scale virtual screening, docking optimization and automated iteration[[109], [110], [111], [112]]. Across the board, the paradigm of large-scale pre-training, task-specific fine-tuning or instruction alignment, followed by generative or retrieval-based reasoning, is now pushing the limits of property prediction, directed evolution, active-site design and ligand discovery, laying a data-driven, model-centric foundation for industrial enzyme engineering.

#### 6.1.6 Contrastive learning-based model

Contrastive learning has recently demonstrated powerful representation-learning capabilities in protein function and structure prediction tasks [113,114]. By constructing positive–negative sample pairs, the method guides the model to learn an embedding space that **captures semantic similarity**; it is widely used in the prediction of the EC number [115], functional classification [116], and structural similarity modeling [116,117].

These AI-based tools can be broadly grouped by their modality (single-modal vs. cross-modal) and their target tasks (e.g., enzyme classification, reaction EC prediction, structural similarity search, or enzyme-substrate matching). Below we highlight representative models from each category.

**Fig. 5:**
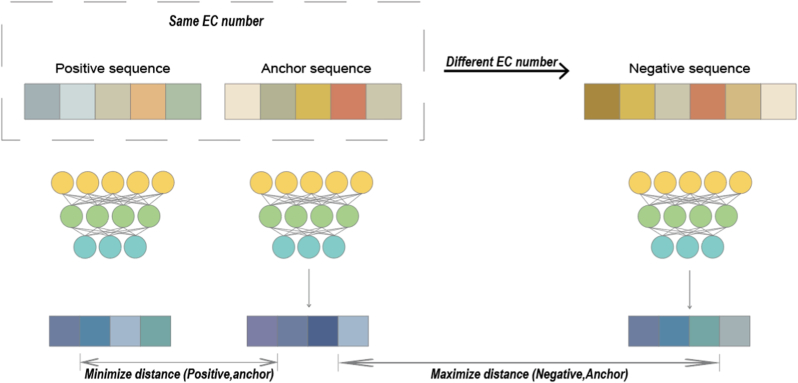
**The contrastive learning–based framework of CLEAN - Train Stage**. The contrastive strategy forces the model to cluster enzymes with identical catalytic functions (same EC class) while separating functionally distinct enzymes (different EC classes) in the latent space. The model minimizes embedding distance between anchor-positive pairs and maximizes embedding distance between anchor-negative pairs. The resulting embeddings encode fine-grained functional relationships transferable to downstream tasks like enzyme function prediction and engineering.

**Fig. 6:**
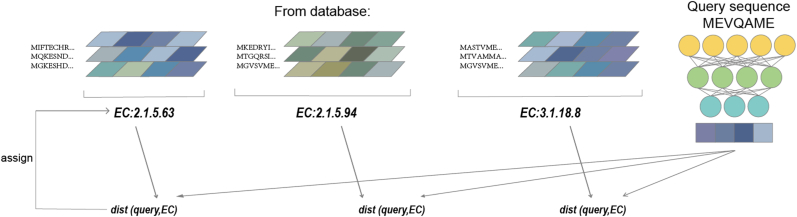
**The contrastive learning–based framework of CLEAN - Predict Stage**. The system processes a query sequence by comparing its learned embedding against precomputed representations of enzyme classes in a reference database. For each EC number category (e.g., 2.1.5.63, 2.1.5.94, 3.1.18.8): Representative enzyme sequences from that functional class are shown. The Euclidean distance between the query embedding and the EC class centroid is calculated. Distances are displayed as dist (query, EC) metric. The model assigns the query sequence to the EC class with the smallest embedding distance, enabling accurate functional prediction [116].

Cross-modal contrastive learning (CLEAN [116], CLAIRE [115]) aligns protein language models (pLMs) with molecular or reaction-graph encoders, markedly improving EC-number classification and signaling the extension of pre-training concepts to multimodal settings. CLEAN [116] introduces a contrastive-learning framework to the enzyme-classification task. By constructing enzyme pairs with identical or different EC numbers, it guides the model to capture functional relatedness(Fig. 5). A protein language model serves as the encoder, and a contrastive loss is applied to its output representations (Fig. 6), thereby enhancing the model's ability to discriminate enzyme functions. CLAIRE [115] is a contrastive-learning model specifically designed for **chemical-reaction EC-number prediction**. Unlike EC-classification models that take protein sequences as input, CLAIRE's input is the **molecular representation of a chemical reaction**, namely, the SMILES strings or molecular graphs of reactants and products. To discover remotely homologous proteins, TM-Vec [98] employs a twin neural network (dual-tower) architecture to enable scalable structurally aware search over protein sequences by predicting TM-score which acts as a metric of structural similarity. To achieve rapid and accurate virtual screening, DrugCLIP [109] uses **contrastive learning** and dense retrieval to train protein-pocket and molecule encoders so that their shared embedding space accurately reflects binding potential.In the field of function prediction for unseen enzymes and enzyme retrieval for new reactions, EnzymeCAGE [97] applies geometry-aware multi-modal architecture and cross-attention between pocket–reaction pairs to align information on unraveling the relationships between enzymes and their catalytic reactions.

In summary, contrastive learning is the core mechanism connecting these models, but their design diverges in terms of input modalities (protein vs. reaction vs. pocket), application scenarios (classification, retrieval, matching), and architecture choices (*e.g.*, dual-tower in TM-Vec vs. cross-attention in EnzymeCAGE), enabling each to address specific challenges in enzyme function prediction.

#### 6.1.7 Diffusion-based models

RF-Diffusion [118] uses an SE(3)-equivariant score-based diffusion process on backbone C*α* coordinates: the forward step adds Gaussian noise in 3-D space, and the reverse step denoises the coordinates back to a folded structure. PiFold [66] substitutes AlphaFold's Evoformer with an SE(3) diffusion module and iteratively denoises backbone frames conditioned on the input sequence. ResGen [95] is a diffusion-probabilistic model applied to ligand atomic coordinates together with pocket atoms; it predicts continuous score functions that guide ligand-atom placement. FoldingDiff [119] implements a DDPM in internal-angle space (six torsion and bond angles per residue) rather than Cartesian space, adding noise to and denoising these angles. DiffDec [120] uses a latent-space diffusion decoder: it first encodes a backbone with a VAE, then runs diffusion in the latent space, and finally decodes the result into an amino-acid sequence. Chai-1 [38] performs pocket-conditioned graph diffusion that incrementally adds atoms and bonds; the reverse process is guided by a pocket-to-ligand distance bias. DiffBP [121] employs hierarchical (coarse-to-fine) diffusion for binding-pose generation: stage 1 predicts a global ligand pose, and stage 2 refines local conformations. DiffSBDD [122] applies diffusion to fragment growing: starting from a core fragment, it diffuses and denoises new fragments while respecting pocket constraints. Boltz-2 [123] combines diffusion sampling with Boltzmann re-weighting, jointly generating ligand poses and binding-affinity scores.

### 6.2 Pre-training era: protein language models

Encoding strategies for reactants and products have progressed from handcrafted descriptors through homology-based evolutionary and similarity features to representations automatically learned by deep-learning models. Large-scale self-supervised pre-training protein language models (pLMs) has fundamentally transformed feature acquisition.

Large-scale self-supervised models such as ESM [101], ProtT5 [124], and ProtBert [125] have demonstrated remarkable capability in encoding protein sequences into contextualised embeddings. These embeddings are widely adopted for downstream property prediction. Pre-trained models serve as out-of-the-box tools that propagate knowledge of proteins and small molecules, downstream tasks can thus learn feature representations more efficiently.

Westlake University's ESM-Ezy [126] fine-tunes ESM-1b on a small but high-quality data set to discover novel multicopper oxidases (MCOs) with outstanding catalytic performance, showcasing the feasibility of using fine-tuned pre-trained models to identify high-performance biocatalysts with low sequence similarity.

UniKP obtains enzyme and substrate embeddings with the ProtT5-XL-UniRef50 model and a SMILES Transformer, respectively, and, using only an extremely randomised tree, achieves efficient prediction of *K*_*M*_,*k*_*cat*_, *k*_*cat*_/*K*_*M*_. LucaOne [127] performs as many as seven biological tasks within a single architecture, which including genus-level taxonomic classification (GenusTax), non-coding RNA family classification (ncRNAFam), protein subcellular localization (ProtLoc), thermostability prediction (ProtStab), antigenicity prediction (InfA), protein–protein interaction (PPI) prediction, and ncRNA–protein interaction (ncRPI) prediction. This multitask learning paradigm not only enhances the model's generality but also strengthens knowledge transfer among tasks.

In addition, ESM-2 [54], ESM3 [36], and Evo 2 [122] conduct general protein pre-training at different scales. Coupled with the SMILES Transformer [99] for small-molecule modeling, they collectively advance molecular-level LLM architectures.

### 6.3 Large-scale multimodal stage: foundation models and agent workflows

With the continuous improvement of large language models (LLMs) in scientific-reasoning ability, an increasing number of studies are integrating them with biomedical tasks to build agent systems endowed with tool invocation and multi-step reasoning. Such systems typically feature a parameterised reasoning paradigm, tool-augmented capabilities, multimodal input understanding, and biological-knowledge integration, enabling them to emulate human scientists’ reasoning more closely and to excel in drug discovery, functional annotation, and molecular-mechanism modeling.

DrugPilot [112] is a LLM-based drug-discovery agent framework that supports multi-step decision-making in complex biomedical tasks. Its key innovation is a parameterised reasoning paradigm that allows users to specify task entities, objectives, and constraints dynamically, thereby constructing controllable, reproducible prompt templates. To enhance knowledge acquisition and verification, DrugPilot integrates multiple external tool plugins, such as the PubChem API, molecule generators, and literature-retrieval interfaces, enabling the LLM to interact with real-world databases. It supports tasks including target identification, molecular-structure design, ADMET property evaluation, and literature-based evidence analysis and generation.

**Fig. 7:**
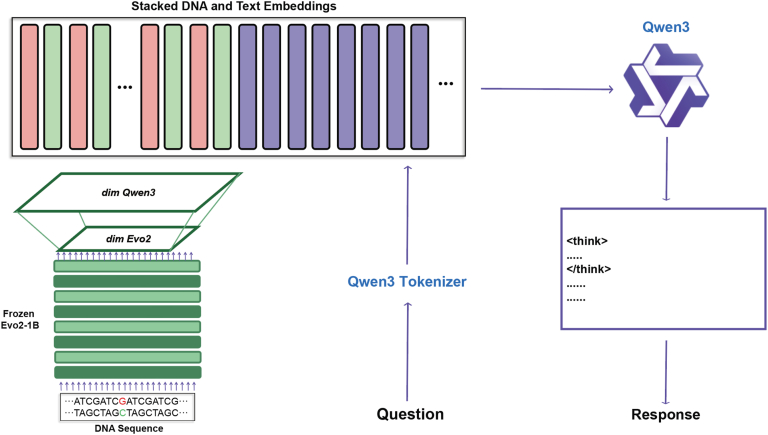
**BioReason Architecture**^c2^: The system combines DNA sequence representations with pretrained biological language model embeddings (Qwen3, Evo2, Frozen Evo2-1B) through dimensional stacking to create fused biological-textual representations. It processes natural language questions using the Qwen3 tokenizer, then conducts multi-step biological reasoning to interpret query context and constraints, translating the refined biological understanding into syntactically and functionally DNA sequence outputs that satisfy the original query requirements. This architecture enables direct conversion of conceptual biological questions into executable genetic designs [52].

BioReason [52] proposes a multimodal biological-reasoning framework that fuses a DNA foundation model (Evo 2) with a general LLM (e.g., Qwen 3), thereby emulating scientists' cross-modal reasoning in gene-mutation analysis and mechanistic modelling. The system takes gene sequences and natural-language queries as its two main inputs, employs Evo 2 to produce context-aware DNA embedding vectors, and combines these embeddings with the LLM's reasoning chain to achieve cross-modal semantic connectivity. A combination of supervised fine-tuning and reinforcement learning (GRPO strategy optimization) improves the scientific soundness of the model's outputs. BioReason can perform tasks such as disease-mechanism reasoning based on KEGG pathways, pathogenicity prediction of gene variants, and generation of functional chains arising from structural mutations(Fig. 7).

## 7 Tasks on enzyme engineering

Enzymes are proteins that possess catalytic activity, making catalysis their fundamental property. One fundamental step in enzyme research is to distinguish enzymes from non-enzymatic proteins. Furthermore, understanding the specific type of biochemical reaction catalyzed by an enzyme is addressed through the prediction of its EC number prediction, which organizes enzymes into a hierarchical classification reflecting their catalytic activity.

Closely related to catalytic function are enzyme kinetic parameters, such as the turnover number, the Michaelis constant, and catalytic efficiency. These parameters characterize enzyme–substrate affinity and catalytic performance, providing quantitative insight into enzymatic performance.

The induced-fit mechanism between enzyme and substrate explains how catalysis occurs, making ligand-binding site (LBS) prediction a crucial task. Besides modeling single-step catalytic reactions, enzyme functions are also classified and represented by Gene Ontology (GO), encompassing Biological Process (BP), molecular function, and cellular component (subcellular localization).

### 7.1 Function modeling

(1) Enzyme–non-enzyme classification.

Only a subset of recent pipelines model this gate explicitly. MVDINET [1] treats it as a de-noising step: non-catalytic proteins are filtered out before its multi-view fusion network proceeds to EC classification. By contrast, most modern contrastive or BERT-style models, *e.g*. CLEAN and CLAIRE, are trained exclusively on curated enzyme datasets; they *assume* the input is catalytic and therefore omit the binary layer. Notably, CLEAN distinguishes itself by learning a representation space where enzyme sequences catalyzing similar reactions cluster together, achieving state-of-the-art accuracy in enzyme function prediction from sequence data alone.

(2) Predicting an EC number.

Efforts to predict enzyme EC numbers have evolved along several complementary lines, utilizing structural, sequence-based, and reaction-centered information. Early pioneering work by Dobson & Doig's 2005 SVM [128] showed for the first time, that coarse 3-D descriptors alone suffice to assign top-level EC classes, bypassing any homology template. Building on this foundation, DEEPre [129] and HECNet integrate sequence, PSSM and predicted structural features with CNN/LSTM or STNet backbones, drilling down to EC levels 2–4. More recently, protein language models combined with contrastive learning have significantly advanced the field. In particular, CLEAN [116] learns a representation space where sequences catalyzing similar reactions cluster, achieving state-of-the-art accuracy from sequence only. CLAIRE [115] extends this idea by pairing the enzyme with its catalyzed reaction graph, boosting level-3/4 recall on low-homology benchmarks. Finally, reaction-aware language models have emerged to explicitly integrate chemical information. BEC-Pred (2024) couples reaction SMILES with enzyme sequences in a dual-input BERT, highlighting the orthogonal mechanistic signal provided by chemistry itself [130].

(3) Enzyme kinetic parameters

Enzyme kinetic parameters typically include the turnover number (*k*_*cat*_), the Michaelis constant (*K*_*M*_), and the catalytic efficiency (*k*_*cat*_/*K*_*M*_).UniKP [15] predicts multiple enzyme kinetic parameters directly from a given enzyme's amino acid sequence and the structural information of its substrate. Moreover, the research team further incorporated environmental factors and proposed an enhanced two-layer framework, EF-UniKP, based on UniKP, to achieve more accurate enzyme kinetic predictions.(Fig. 4). DLKcat predicts *k*_*cat*_ by converting substrate molecules into graphs using graph neural networks (GNNs), where nodes represent atoms and edges represent chemical bonds. It adopts an *r*-radius subgraph and bidirectional vertex/edge updates to capture localized chemical environments. For enzymes, it uses convolutional neural networks (CNNs), segmenting protein sequences into 1–3-g words, embedding them, and extracting local patterns through convolution followed by ReLU activation. These convolutional layers are stacked to enhance feature representation (Fig. 3).

(4) GO annotation

GO annotation, also known as Automatic Protein Function Prediction (AFP), aims to predict hierarchical, multi-label Gene Ontology (GO) terms from three ontologies: Biological Process (BP), Molecular Function (MF), and Cellular Component (CC), for a given protein. It is characterized as a hierarchical, multi-label, and extremely long-tailed classification task. The primary evaluation benchmark is provided by the Critical Assessment of Function Annotation (CAFA) challenge. NetGO [131] incorporates large-scale pretrained language models (pLMs), such as ESM-2, into its framework. DeepGO-SE [132] converts GO axioms into logical statements, leveraging pLMs as fact-checking evaluators to achieve function prediction from a semantic entailment perspective. ProtGO [133] adopts an end-to-end Transformer architecture, while ProtCLIP [134] employs multimodal pretraining based on sequence-text alignment, significantly improving GO-CC and GO-BP prediction by contrasting UniProt entries against titles and abstracts of scientific literature.

(5) Binding-site prediction

Catalysis requires induced fit between enzyme and substrate; hence residue-level LBS prediction is a vital companion task. Geometry-aware CNNs (DeepSite [135], DeepPocket [136]), equivariant GNNs (ScanNet [137], PeSTo [138]) progressively refine pocket localization, providing structural priors that can be fed back into EC or mechanism predictors.

(6) Cofactor specificity prediction

Cofactors are non-protein small molecules or ions that bind to and activate enzyme proteins, enabling catalytic activity, enhancing catalytic efficiency, increasing substrate specificity, and expanding the range of enzymatic reactions. Among them, FAD typically forms a stable complex with enzymes through covalent or non-covalent interactions and is regarded as an integral part of the enzyme's structure, making it suitable for structure-based prediction. In contrast, NAD/NADH participates transiently in reactions in a free form, functioning more like a substrate or product in enzymatic catalysis. As such, their interactions with enzymes are usually treated as enzyme–substrate interactions and are not suitable to be modeled as fixed cofactors in structure-based prediction.

These cofactor-related AI tools can be categorized by their modeling focus: structure-based prediction (*e.g.,* MaSIF, AlphaFill), sequence-based classification (*e.g.,* Cofactory, DISCODE, Logistic), or integrated design frameworks (*e.g*., Rossmann-toolbox). Below we highlight representative methods across these categories.

Cofactory (2014) [87] employs a combination of structural alignment (MUSTANG) and HMMER to construct profile HMMs for each cofactor type. Based on the aligned sequences of Rossmann-fold domains, the method uses three separate feedforward neural networks to predict whether a given domain binds to FAD, NAD, or NADP, respectively. MaSIF (2020) [139] proposes a geometric deep learning framework based on protein molecular surfaces to learn functional interaction fingerprints. A convolutional network processes spherical surface patches to extract surface embeddings, which can be applied to downstream tasks such as protein–small molecule recognition, protein–protein interaction prediction, and cofactor-binding site analysis. MaSIF-site predicts potential binding sites on protein surfaces by assigning binding probabilities to each surface point based on local surface fragments. MaSIF-search rapidly compares protein surfaces to identify complementary matching regions, outputting similarity scores and matched surface points. MaSIF-ligand is used to distinguish between different types of small molecule ligands (such as cofactors, ATP, *etc*.). Rossmann-toolbox (2022) [140] supports both prediction and design tasks. For prediction, given the *β* − *α* − *β* fragment sequence from a protein structure, it employs a multi-layer Bi-LSTM with fully connected layers to predict which cofactor is bound, supporting four types: NAD, NADP, FAD, and SAM. For design, given a target cofactor, the model recommends specific residue mutations to alter the protein's native cofactor preference toward the desired type. AlphaFill (2023) [34] supports the prediction of 12 types of cofactors by enriching AlphaFold-predicted structures through template-based database matching and structural superposition. Logistic(2022) [141] focuses on high-resolution cofactor specificity prediction between NAD and NADP, using logistic regression to score residue-level contributions and identify key determinants of specificity. DISCODE(2025) [142] (Deep learning-based Iterative pipline to analyze Specificity of COfactors and to Design Enzyme) is a transformer-based NAD/NADP classification model. The model uses ESM-2 language model for amino acid embedding, finally represents the probability of NAD and NADP. This code can also be run on Google Colaboratory.

In summary, structure-based models (*e.g.*, MaSIF, AlphaFill) excel at capturing spatial and physicochemical properties of cofactor binding, while sequence-based models (*e.g*., Cofactory, DISCODE, Logistic) are lightweight and suitable for large-scale screening. Integrated approaches like Rossmann-toolbox offer both classification and design guidance, supporting practical enzyme re-engineering.

### 7.2 Structure modeling

Modeling the structures of molecules such as enzymes, substrates, and DNA/RNA forms the foundation for extracting structural information to enable tasks such as pocket-based substrate prediction, small molecule generation, and d*e novo* enzyme design. Structure modeling can be categorized into two types: (1) Monomer modeling of individual proteins, substrates, or cofactors; (2) Structure prediction and d*e novo* design of complexes, such as protein-ligand interactions, protein-DNA/RNA interactions, and protein-protein interactions.

#### 7.2.1 Monomer prediction

Monomer structure prediction has advanced from traditional fragment-based or template-based modeling to end-to-end deep-learning frameworks. Pioneering models such as Chai-1 [38], AlphaFold 3 [35], and RoseTTAFold All-Atom [143] have demonstrated the ability to accurately predict single-chain protein structures directly from sequence, while also generalizing to handle small-molecule cofactors. These models integrate physical constraints, geometric priors, and evolutionary information in a unified architecture, pushing monomer prediction toward near-experimental resolution. Such advances lay the foundation for downstream tasks including functional annotation, stability estimation, and rational design. The following structure-based inverse folding models can be grouped by their sequence generation strategy (autoregressive vs. one-shot), representation form (3D grid, graph, *etc.*), and design objectives (general sequence design vs. interface optimization).

ProteinMPNN [144] operates on a 3D backbone graph using multi-layer message passing to aggregate local structural information. This enables sequence optimization on existing backbones to improve properties such as stability, expression yield, or binding affinity, and allows the design of protein–protein interfaces with maximized binding energy or specificity. DenseCPD [145] is a structure-based computational protein design method. Its core idea is to model the three-dimensional spatial distribution of backbone atoms surrounding a target residue. For each target residue in the protein structure, DenseCPD aligns the C*α* atom of the residue to the origin and orients the C*β* atom along the positive z-axis, constructing a cubic grid of size 20 Å × 20 Å × 20 Å. This grid is used to capture the spatial density information of N, C*α*, C, C*β*, and O atoms in the neighborhood of the target residue, with each atom type corresponding to one channel, resulting in a total of five channels. PiFold [66] breaks away from the autoregressive generation paradigm by introducing PiFold, a model equipped with highly expressive structural features and an efficient PiGNN module. It adopts a one-shot generation strategy to generate the entire protein sequence in a single step, significantly improving both the accuracy and efficiency of protein sequence design. Compared to methods such as StructGNN [146], ProteinMPNN [144], ESM-IF, and Structured Transformer [147], PiFold achieves notable improvements, attaining state-of-the-art (SOTA) performance on the **CATH4.2**, **TS50**, and **TS500** datasets. In comparison, ProteinMPNN and PiFold both utilize graph representations but differ in generation strategy: PiFold's one-shot decoding significantly boosts speed while maintaining accuracy. DenseCPD, on the other hand, offers interpretable residue-level modeling via voxel grids. Overall, these tools provide complementary solutions for tasks ranging from stability-oriented sequence redesign to interface-specific optimization.

The protein sequence generation model LM-Design [105] consists of a structure encoder and a sequence decoder. The structure encoder is a pretrained graph neural network that encodes protein structures, while the sequence decoder is based on a large-scale pretrained protein language model, resembling a BERT/Transformer encoder with bidirectional self-attention, and incorporates a structural adaptor at the final layer. In terms of efficiency, LM-Design further surpasses PiFold. In addition, new methods such as AMPLIFY [57] and RFAA have further explored conformation-controllable protein design, pushing structure prediction models toward the capability of performing customizable conformational transitions.

#### 7.2.2 Complex interaction prediction

Complex interaction prediction focuses on modeling the structures and mechanisms of biomolecular assemblies, including protein–protein, protein–DNA, protein–RNA, and protein–ligand.

**Fig. 8:**
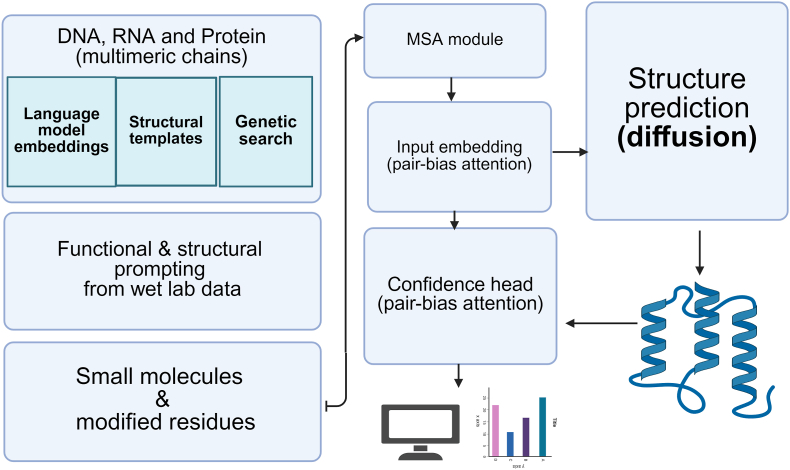
Overview of Chai-1. Chai-1 accepts a wide variety of optional input features, such as language model embeddings, structural templates, genetic search, and wet-lab experimental data such as contacts determined by cross link mass spectrometry or epitope mapping.

In the field of **molecular interaction prediction between proteins and nucleic acids**, models such as AlphaFold 2 [148], AlphaFold 3 [42], RoseTTAFold All-Atom [143], and Chai-1 [38] have demonstrated the capability of modeling multi-molecule coupled systems(Fig. 8). Protenix supports structure modeling for protein–ligand, protein–nucleic acid, and antibody–antigen interactions [149].

In the task of **predicting compound–protein interactions (CPI)**, a broad range of generative and discriminative models have emerged. For example, TransformerCPI2.0 [107] utilize a Transformer-based architecture to discover new hits, while other methods such as DrugGPT [150], libpqr [151], AlphaDrug [152], Pocket2Mol [153], ResGen [95], SurfGen [96], DiffDec [120], PocketFlow [154], TargetVAE [155], LiGAN [156], and RGA [157] incorporate graph-based, flow-based, or genetic strategies to optimize candidate compounds for target binding.

### 7.3 Property modeling

The functional performance of enzymes is affected by various properties of proteins and their complexes with other components such as substrates. These properties include stability, thermostability, and salt tolerance. EF-UniKP enables robust prediction of *k*_*cat*_ values while taking into account environmental factors such as pH and temperature [15]. ProtSSN represents a structure-informed approach for predicting the fitness impact of mutations on catalytic activity and thermostability. It aligns sequence–structure–function modeling with practical needs in protein engineering, and has been shown to effectively support the enhancement of protein thermostability [6].

## 8 Challenge and outlook

Although AI technologies and models have found numerous applications in enzyme engineering, several tasks remain insufficiently addressed. Moreover, we have identified several emerging trends in AI for enzyme engineering, some are on the verge of becoming industry consensus, while others hold significant promise for future development.

### 8.1 From encoder-centric modeling to foundation model fine-tuning

Despite substantial progress, several enzyme-engineering tasks remain insufficiently addressed by existing AI approaches. As the field matures, we identify emerging trends: some approaching consensus, others holding transformative potential.

At the core of these developments lies a shift from task-specific, end-to-end modeling toward the use of pretrained foundation models that serve as universal encoders. These models, such as ESM-2 and Evo, extract rich, transferable representations from protein sequences, which can be fine-tuned for diverse downstream applications, including enzyme classification, cofactor prediction, *De novo* design, and protein-ligand interaction modeling.

For instance, UniKP employs a modular architecture with a dedicated encoder for representation learning and a decoder for task-specific inference. Similarly, ProtSSN and ProteinF3S leverage semantic embeddings generated by ESM-2 to enhance predictive performance across enzyme-related tasks [6].

The ESM model family exemplifies this trend, employing transformer-based architectures to integrate information from single sequences, multiple sequence alignments (MSAs), residue-residue contact maps, and secondary structural features [54,92]. Parallel innovations such as Token-Mol introduce fully discrete molecular representationslike transforming SMILES strings, torsion angles, and chemical properties into tokenized formats compatible with large language models (LLMs). This enables seamless integration of chemical data with LLMs and paves the way for multimodal learning systems.

Looking forward, we anticipate a bifurcation in research efforts: (i) The race to develop powerful encoder-based foundation models optimized for protein embedding; (ii) The proliferation of fine-tuned downstream decoders that repurpose these embeddings across diverse enzyme-design pipelines.

### 8.2 From task-specific small models to versatile multi-task foundation models

Thanks to years of accumulated database resources and foundational work in sequence, structure, and function modeling, the field has witnessed a shift from specialized models to increasingly general-purpose and powerful systems. Starting with AlphaFold, which focuses on d*e novo* protein structure prediction, the development has progressed to ESM3 [36], which jointly models sequence, structure, and function. This evolution continued with Evo2 [111], capable of genome-scale modeling and generation, and further advanced to BioReason [52], which integrates Evo2's powerful sequence representation capabilities with Qwen3 [158]'s multi-step reasoning abilitieswhich enabling direct processing of genomic information as input for downstream reasoning.

These developments illustrate a clear trend: models are becoming increasingly large-scale and versatile, moving from task-specific small architectures to unified platforms capable of handling diverse tasks across the entire enzyme engineering pipeline.

### 8.3 From automated research tools to autonomous scientific discovery agents

AI is rapidly evolving from a passive tool assisting biologists to an active, even autonomous, scientific collaborator capable of exploring unknown biological spaces. Recent developments illustrate this trend clearly. For example, SYMPLEX, a knowledge extraction platform for synthetic biology enabled by large language models, focuses on knowledge discovery in fundamental research [159]. Similarly, VENUSFACTORY [160] developed in 2025 b y a joint team from Shanghai Jiao Tong University, Shanghai Artificial Intelligence Laboratory, and East China University of Science and Technology. VenusFactory is a unified platform designed for protein engineering that integrates biological data retrieval, standardized task benchmarking, and modular fine-tuning of pretrained protein language models (pLMs). The platform supports both command-line execution and a no-code interface based on Gradio, incorporating over 40 protein-related datasets and more than 40 popular pLMs, making it accessible to researchers in both computer science and biology.

DrugPilot [112] combines large language models (LLMs) with a parameterised reasoning mechanism to support the drug discovery pipeline with automation, multi-stage coordination, and high precision. It addresses key challenges such as multimodal data processing, context loss, and reasoning bias. The system is capable of completing the full workflow from molecule generation, encompassing molecule generation, property prediction, drug optimization, and synthesis pathway design.

BioReason [52] enables cross-modal semantic reasoning through its reasoning chain. By combining supervised fine-tuning and reinforcement learning (GRPO strategy optimization), it enhances the scientific soundness of model outputs. It performs tasks such as disease mechanism inference based on KEGG pathways, pathogenicity prediction of gene variants, and generation of functional chains arising from structural mutations.

### 8.4 From static structure modeling to dynamic folding pathway modeling

At present, protein structure prediction and function modelling remain dominated by static approaches, i.e. algorithms that infer a single, stable end-state conformation from a given amino-acid sequence or, conversely, generate sequences expected to fold into a target fold. Two methodological paradigms prevail: graph-neural-network (GNN)-based structural modelling and protein-language-model (PLM)-based sequence modelling. Within these frameworks a wealth of landmark studies has appeared. The AlphaFold and RoseTTAFold families (including AlphaFold3 and RoseTTAFold2) have achieved near-atomic accuracy in end-to-end *sequence-to-structure* inference, sparking a revolution in structural biology. In structure-conditioned sequence generation, GNN-style decoders such as ProteinMPNN [144], LigandMPNN [161] and HighMPNN [162] excel, while ESM-IF1, AlphaDesign and QDF [163] explore structure-guided sequence optimization strategies. Methods such as GraphDTA (GAT-GCN), CPI-GNN, TransformerCPI 2.0 and MolTrans couple ligand graphs and protein representations through GNNs or Transformer backbones to predict binding affinity or activity. Models including ESM, ProtBert, ProtT5, ProtGPT-2 and DrugGPT employ a pre-train-fine-tune paradigm to learn the *semantics* of protein sequences, delivering state-of-the-art performance in classification, mutational-effect prediction and functional annotation. Despite their widespread success, all of the above methods rely on a single end-state structure or static sequence features. They cannot capture the rich ensemble of intermediate states, alternative folding routes or kinetic behaviours that real proteins explore. Consequently, deciphering and modelling the folding pathway dynamics constitutes both the major challenge and the natural next stage in protein modelling.

Artificial intelligence methods have transformed static structure prediction, yet the intrinsic dynamics of proteins has long been under-represented. Molecular-dynamics (MD) simulations already open a window onto those dynamics, and recent large-scale MD trajectory repositories lower the data barrier; what is still missing are efficient representations of time-resolved information. The Vitalis group from University of Zurich addressed this gap with Nearl [68], a toolkit that encodes MD trajectories into 3-D voxelised features directly consumable by neural networks. Nevertheless, mainstream *inverse-folding* methods (e.g. ProteinMPNN) do not yet solve the genuine problem articulated by Sergey — finding sequences that fold uniquely into a desired structure while avoiding all competing conformations. Achieving that goal requires explicit modelling of the folding-energy landscape and the manifold kinetic routes across the conformational space. The critical bottleneck is the near-absence of experimental folding-trajectory data suitable for supervised learning. Building a high-throughput platform that can record folding events at atomic spatial and sub-millisecond temporal resolutionby combining NMR spectroscopy, optical tweezers and ultrafast imaging, would be strategically transformative.

Ultimately, we aim for AI models that infer the entire folding movie from sequence alone: not merely the final structure, but the full trajectory and energy landscape. Such models would enable the rational design of sequence-structure systems endowed with bespoke dynamics and energetic profiles, ushering in a new era of precision protein engineering.

## 9 Conclusion

This review systematically summarizes the applications of AI in enzyme engineering tasks related to function, structure, and property modeling. It outlines the technological evolution from traditional machine learning approaches to large-scale pretrained model paradigms, and provides an integrated reference framework for newcomers and interdisciplinary researchers in the field.

We highlight that AI models are transitioning from independent, task-specific modeling to unified frameworks powered by foundation models, evolving from static structure prediction toward dynamic behavior simulation, and gradually developing into intelligent agent systems capable of reasoning. These trends signal a profound shift in the research paradigm of enzyme engineering.

Future research should focus on the following directions: (1) establishing standardized benchmark datasets to enhance model transferability and evaluation across tasks; (2) integrating temporal dynamics and multimodal priors to develop unified frameworks for structure–function modeling; (3) improving model interpretability and coupling with experimental validation to increase reliability in real-world applications; and (4) advancing the integration of foundation models with domain-specific tools to build intelligent agent platforms capable of autonomous design, reasoning, and laboratory interaction.Ultimately, we envision a future where AI not only accelerates enzyme engineering workflows, but also enables entirely new modes of scientific discovery: transforming proteins from passive molecular tools into actively designed, dynamic systems tailored with precision for increasingly complex and ambitious biotechnological applications.

## Author contributions

Haiyang Cui conceived of the structure of this review. Ziyan Shi, Shuping Xu, and Sihan Xue wrote the main content of the original draft. Kaiming Chen and Yifan Lu She drew part of the figures. Yannan Tian, Yanhui Gu, Junsheng Zhou, Shuaiqi Meng, Hao Zhou, Yuefei Wang, Siyu Long, Jianing Wang, Peng Zhang participated in the discussion of the article and contributed professional guidance. Haiyang Cui and Shuaiqi Meng reviewed and revised the manuscript. Haiyang Cui provided supervision, funding, and resources for this study.

## Declaration of competing interest

The authors declare that they have no competing interests.
